# Mitochondrial and endoplasmic reticulum stress pathways cooperate in zearalenone-induced apoptosis of human leukemic cells

**DOI:** 10.1186/1756-8722-3-50

**Published:** 2010-12-30

**Authors:** Ratana Banjerdpongchai, Prachya Kongtawelert, Orawan Khantamat, Chantragan Srisomsap, Daranee Chokchaichamnankit, Pantipa Subhasitanont, Jisnuson Svasti

**Affiliations:** 1 Department of Biochemistry, Faculty of Medicine, Chiang Mai University, Chiang Mai 50200, Thailand; 2 Laboratory of Biochemistry, Chulabhorn Research Institute, Bangkok 10210, Thailand; 3 Department of Biochemistry, Faculty of Science, Mahidol University, Rama VI Road, Bangkok 10400, Thailand

## Abstract

**Background:**

Zearalenone (ZEA) is a phytoestrogen from *Fusarium *species. The aims of the study was to identify mode of human leukemic cell death induced by ZEA and the mechanisms involved.

**Methods:**

Cell cytotoxicity of ZEA on human leukemic HL-60, U937 and peripheral blood mononuclear cells (PBMCs) was performed by using 3-(4,5-dimethyl)-2,5-diphenyl tetrazolium bromide (MTT) assay. Reactive oxygen species production, cell cycle analysis and mitochondrial transmembrane potential reduction was determined by employing 2',7'-dichlorofluorescein diacetate, propidium iodide and 3,3'-dihexyloxacarbocyanine iodide and flow cytometry, respectively. Caspase-3 and -8 activities were detected by using fluorogenic Asp-Glu-Val-Asp-7-amino-4-methylcoumarin (DEVD-AMC) and Ile-Glu-Thr-Asp-7-amino-4-methylcoumarin (IETD-AMC) substrates, respectively. Protein expression of cytochrome c, Bax, Bcl-2 and Bcl-xL was performed by Western blot. The expression of proteins was assessed by two-dimensional polyacrylamide gel-electrophoresis (PAGE) coupled with LC-MS2 analysis and real-time reverse transcription polymerase chain reaction (RT-PCR) approach.

**Results:**

ZEA was cytotoxic to U937 > HL-60 > PBMCs and caused subdiploid peaks and G1 arrest in both cell lines. Apoptosis of human leukemic HL-60 and U937 cell apoptosis induced by ZEA was via an activation of mitochondrial release of cytochrome c through mitochondrial transmembrane potential reduction, activation of caspase-3 and -8, production of reactive oxygen species and induction of endoplasmic reticulum stress. Bax was up regulated in a time-dependent manner and there was down regulation of Bcl-xL expression. Two-dimensional PAGE coupled with LC-MS2 analysis showed that ZEA treatment of HL-60 cells produced differences in the levels of 22 membrane proteins such as apoptosis inducing factor and the ER stress proteins including endoplasmic reticulum protein 29 (ERp29), 78 kDa glucose-regulated protein, heat shock protein 90 and calreticulin, whereas only *ERp29 *mRNA transcript increased.

**Conclusion:**

ZEA induced human leukemic cell apoptosis via endoplasmic stress and mitochondrial pathway.

## Introduction

The phytoestrogen zearalenone (ZEA) is one of the most active naturally occurring estrogenic compounds [1,2]. Food, snacks, dried fruits, dried vegetables and beverages such as beer, often contain ZEA [3-5]. The average daily intake of ZEA in adults ranges from 0.8-29 ng/kg body weight (b.w.)/day, while small children have a higher average daily intake, 6-55 ng/kg b.w./day [6].

Treatment with Zea (10-40 μM) of Vero, Caco-2 and DOK cells results in apoptosis as evidenced by DNA ladder formation and presence of apoptotic bodies [7]. Recently, ZEA has been shown to induce apoptosis in human hepatocytes (HepG2) via p53-dependent mitochondrial signaling pathway with the up regulation of ATM and GADD45 involved in DNA repair [8].

In mammalian cells, there are two major pathways involved in apoptosis: mitochondria-initiated intrinsic pathway and death receptor-stimulated extrinsic pathway [9-11]. In the former pathway, proapoptotic signals provoke release from mitochondrial inter-membranous space into cytosol of cytochrome c, which forms a complex with Apaf-1 and dATP, known as apoptosome, and triggers caspase-9 activation. Activation of caspase-9 leads to subsequent activation of executioner caspases, such as caspase-3, -6, -7, which in turn stimulates a series of apoptotic events, eventually leading to cell death [9,12,13]. The extrinsic pathway begins with binding of Fas ligand to Fas death receptor, and an adaptor molecule is recruited to the receptor, which allows binding and proteolytic activation of caspase-8. Activated caspase-8 then cleaves effector caspase-3, -6 and -7, leading to apoptotic cell death [10,12,14].

In addition to the above mentioned pathways, apoptosis can be induced via endoplasmic reticulum (ER), which normally regulates protein synthesis and intracellular calcium (Ca^2+^) homeostasis [15]. Excessive ER stress triggers apoptosis through a variety of mechanisms including redox imbalance, alteration in Ca^2+ ^level and activation of Bcl-2 family proteins [16].

Calreticulin (CRT) is an abundant Ca^2+^-binding chaperone, which is mostly present in ER lumen, although it can also be found in other subcellular localizations [17,18]. When present on the surface of damaged cells, it can serve as an 'eat-me' signal and hence facilitates the recognition and later engulfment of dying cells by macrophages [19] or by dendritic cells [20]. It is thought that this function determines the immunostimulatory effect of CRT, as presentation of tumor antigens by dendritic cells is required for the immunogenic effect of anthracyclin-treated cancer cells [20-22]. Alternatively, CRT may bind tumor antigenic peptides and facilitate their efficient presentation to T cells [23]. Crosstalk with the two well-characterized apoptotic pathways also exists, since ER stress can also activate caspase-8 and caspase-9 [24,25].

The ability of ZEA to modulate leukemic cell growth has not yet been well characterized. Using two human leukemic HL-60 and U937 cell lines we found that human leukemic cell apoptosis induced by ZEA was related to caspase-3 and -8 activation, mitochondrial transmembrane potential (MTP) reduction and cytochrome c release. ZEA also induced oxidative stress via ROS generation, Bax upregulation and Bcl-xL downregulation. The mechanistic effect also involved increased Ca^2+ ^concentration in cytosol and mitochondria indicating ER stress but there was no calreticulin exposure on the cell surface at 30 min. Two-dimensional gel-electrophoresis of proteins following 24 h treatment revealed upregulated expression of ER-mediated chaperone endoplasmic reticulum protein 29 (ERp29), 78 kDa glucose regulated protein (GRP78), and calreticulin supporting the involvement of ER stress.

## Materials and methods

### Chemicals and test media

**Figure 1:**
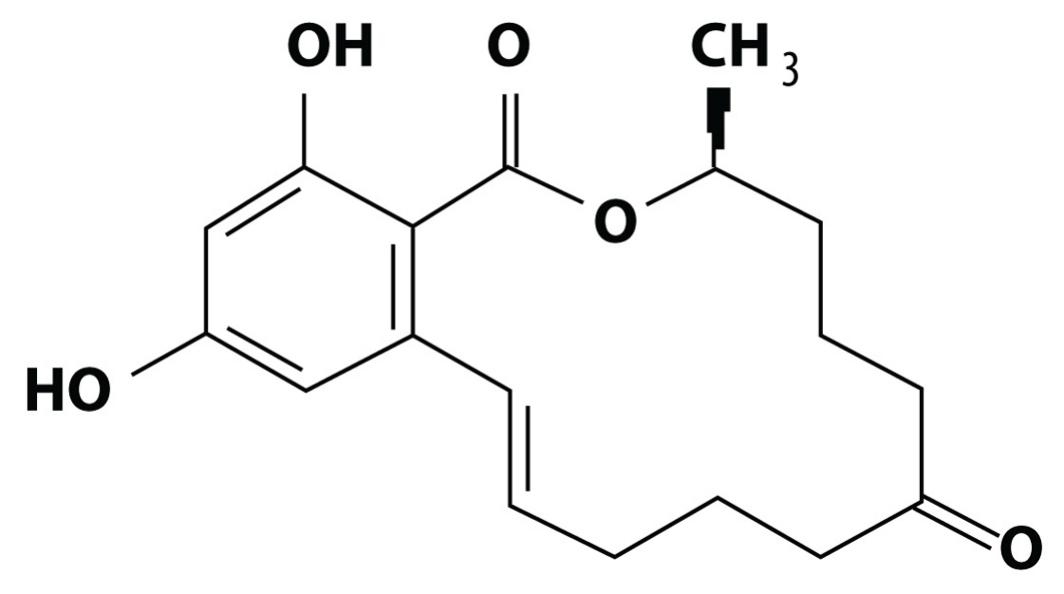
Structure of zearalenone (ZEA; 6-[10-hydroxy-oxo-trans-1-undecenyl]-B-resorcyclic acid lactone).

ZEA (Figure 1), lymphoprep, MTT (3-(4,5-dimethyl)-2,5-diphenyl tetrazolium bromide, propidium iodide (PI), 3,3'-dihexyloxacarbocyanine iodide (DiOC_6_), 2',7'-dichlorofluorescein diacetate (DCFH-DA), ProteoExtract Albumin/Removal kit, and ProteoPrep Universal Protein Extraction kit were obtained from Sigma-Aldrich (St. Louis, MO, USA). RPMI-1640 medium, SYBR GREENER qPCR UNIVERSAL and primers sequences were obtained from Invitrogen, USA. DEVD-AMC (Asp-Glu-Val-Asp-7-amino-4-methylcoumarin) and IETD-AMC (Ile-Glu-Thr-Asp-amino-4-methylcoumarin) were obtained from Biosource, USA. IPG gel strips were purchased from GE Healthcare, Uppsala, Sweden. Trypsin was obtained from Promega Madison, WI, USA. Mouse monoclonal antibodies to cytochrome c, Bax and Bcl-2 and rabbit polyclonal antibody to Bcl-xL, and horseradish peroxidase (HRP) conjugated secondary antibodies were purchased from Abcam, Cambridge, UK. SuperSignal West Pico Chemiluminecent Substrate was obtained from Pierce, Rockford, IL, USA. Complete mini protease inhibitor cocktail was obtained from Roche, Basel, Switzerland. Fluo3-AM and Rhod2-AM were obtained from Molecular Probes, Eugene, OR, USA. RNA extraction kit was obtained from Pharmacia Bioscience, Uppsala, Sweden. RevertAid™ First Strand cDNA Synthesis kit was obtained from MBI Fermentas, Germany.

### Cell culture

Human promyelocytic leukemic HL-60 and human promonocytic U937 cells were gifts from Dr. Sukhathida Ubol and Dr. Watchara Kasinroek. The cells were cultured in 10% fetal bovine serum in RPMI-1640 medium supplemented with penicillin G (100 units/ml) and streptomycin (100 μg/ml) at 37°C in a humidified atmosphere containing 5% CO_2_. The human leukemic cells (1 × 10^6^) were treated with ZEA at indicated concentration and duration. ZEA was dissolved in DMSO as a vehicle and the maximal volume used was not exceeded 10 μl/ml of media.

The blood was obtained from adult volunteers with IRB approval. Peripheral blood mononuclear cells (PBMCs) were isolated from heparinized blood by density gradient centrifugation using lymphoprep according to standard protocols. Cells were cultured in RPMI-1640 medium supplemented with 10% heat-inactivated fetal bovine serum, 2 mM glutamine, 100 U/ml penicillin and 100 μg/ml streptomycin. PBMCs (3 × 10^6^) were treated with ZEA at indicated concentration and duration.

### Cytotoxicity test

Following ZEA treatment, cell viability was assessed by MTT (3-(4,5-dimethyl)-2,5-diphenyl tetrazolium bromide) assay [26]. This method is based on the ability of viable cells to reduce MTT and form a blue formazan product. MTT solution (sterile stock solution of 5 mg/ml) was added to cell suspension at a final concentration of 100 μg/ml and the solution incubated for 4 h at 37°C in a humidified 5% CO_2 _atmosphere. The medium was then removed and cells were treated with DMSO for 30 min. The optical density of the cell lysate was measured at 540 nm with reference wavelength of 630 nm using microtiter plate reader (Biotek, USA). Number of viable cells was calculated from untreated cells, and the data were expressed as percent cell viability.

### Determination of mitochondrial transmembrane potential and ROS production

For measurement of mitochondrial membrane potential and intracellular ROS, either 40 nM 3,3'-dihexyloxacarbocyanine iodide (for mitochondrial transmembrane potential determination) or 5 μM 2',7'-dichlorofluorescein diacetate (for ROS detection) were added for 15 min at 37°C and the cells are then subjected to flow cytometry.

For flow cytometric assessment of DNA fragmentation and cell cycle distribution, 1 × 10^6 ^cells were harvested and re-suspended in a solution containing PI (50 μg/ml), 0.1% Triton X-100 and 0.1% sodium citrate in PBS. Cells then were analyzed in a FACScan equipped with a 488 nm argon laser using CellQuest software (Becton-Dickinson, USA). Data were depicted as histograms and the percentage of cells displaying hypodiploid DNA content was indicated. Percentage of cells in each phase was also evaluated to determine the existence of cell cycle arrest.

### Assay of caspase-3 and caspase-8 activity

Cleavage of the fluorogenic peptide substrates DEVD-AMC and IETD-AMC, indicative of caspase-3-like and caspase-8-like enzyme activity, was estimated. Cell lysates (1 × 10^6 ^cells) and substrate (50 μM) were combined in a standard reaction buffer and added to a 96-well plate. Enzyme-catalyzed release of AMC was measured by a fluorescence plate reader (Bio-tek, USA) using 355 nm excitation and 460 nm emission wavelengths.

### Two-dimensional polyacrylamide gel-electrophoresis (2-D PAGE)

U937 cells, treated and untreated with 20 μM ZEA for 4 and 24 h were harvested and washed twice and the cell precipitates were used further. Albumin was first removed using ProteoExtract Albumin/Removal kit. The amount of protein loaded in 2-D PAGE was 200 μg/gel. 2-D PAGE was performed using the immobiline/polyacrylamide system. Samples were applied by overnight in-gel rehydration of 70 mm nonlinear pH 3-10 IPG gel strips. The first dimension (IEF) was performed at 6500 Vh for 3.5 h, using a Pharmacia LKB Multiphor II system. IPG strips were equilibrated with buffer in two steps. The first step employed 50 mM Tris-HCl buffer, pH 6.8, 6 M urea, 30% glycerol, 1% SDS, and 1% DTT, while 2.5% iodoacetamide replaced DTT in the second step. Then IPG strips were applied to the second-dimension 12.5% T SDS polyacrylamide gels (100 mm × 105 mm × 1.5 mm). Electrophoresis was performed in a Hoefer system at 20 mA for 2.5 h at room temperature. After electrophoresis, proteins were visualized by CBR-250 staining.

### PAGE of plasma membrane proteins

ProteoPrep Universal Protein Extraction kit was used to isolate membrane and cytosolic proteins from HL-60 cell line. The cytoplasmic extraction reagent was added to the cell pellet and the sample was sonicated at 4°C and centrifuged at 14,000 × *g *for 45 min. The supernatant was collected. The same reagent was added to the remaining pellet, followed by sonication and centrifugation, and the resulting supernatant was pooled with that obtained earlier. The pooled supernatant was dried using Speed Vac. The dried sample was resuspended in the soluble protein resuspension reagent (Sup1).

The precipitate was resuspended in cellular and organelle membrane solubilizing reagent. The sample was centrifuged at 14,000 × *g *for 45 min at 15°C. The supernatant was collected as Sup2. Sup1 and 2 were treated with 5 mM tributylphosphine (TBP) (reduction) for 1 h at room temperature, then 15 mM iodoacetamide (alkylation) was added and the reaction mixture was incubated for 1.5 h. The reaction was stopped by adding TBP and incubated for 15 min. The sample was centrifuged at 20,000 × *g *for 5 min at room temperature and the clear supernatant was collected. The concentrations of proteins in Sup1 and Sup2 were measured using the Bradford method. Samples were prepared for 2-D PAGE by adding ampholine and solubilizing reagent to adjust the volume.

2-D PAGE was performed using the immobiline/polyacrylamide system. Samples were applied by overnight in-gel rehydration of 70 mm nonlinear pH 3-10 IPG gel strips. The first dimension electrophoresis (IEF) was performed as described for U937 cells.

### Tryptic in-gel digestion of protein spots

Differential expression of proteomic profiles in treated and untreated cell lines were compared. Spots of interest were excised and transferred to 1.5 ml tubes. A 50 μl aliquot of 0.1 M NH_4_HCO_3 _in 50% acetonitrile was added, and the gel was incubated for 20 min at 30°C. The solvent was discarded and the gel particles were dried completely. Reduction and alkylation was performed by swelling the gel pieces in 50 μl buffer solution (0.1 M NH_4_HCO_3_, 10 mM DTT, and 1 mM EDTA) and incubating at 60°C for 45 min. Then the excess liquid was removed and quickly replaced by the same volume of freshly prepared 100 mM iodoacetamide in 0.1 M NH_4_HCO_3 _solution. The gel suspension was incubated at room temperature in the dark for 30 min and iodoacetamide solution removed. Each gel piece was washed with 50% acetonitrile in water 3 times for 10 min, and completely dried. A 50 μl aliquot of digestion buffer (0.05 M Tris HCl, 10% acetonitrile, 1 mM CaCl_2_, pH 8.5) and 1 μl aliquot of trypsin (1 mg trypsin in 10 μl 1% acetic acid) were added to the gel pieces. The mixtures were incubated at 37°C overnight. The digestion buffer was removed and saved. The gel pieces were then extracted by adding 60 μl of 2% freshly prepared trifluoroacetic acid and incubating for 30 min at 60°C. The extract and saved digestion buffer were pooled and dried. Digested peptides were dissolved in 6 μl of 0.1% formic acid for MS/MS injection.

### Protein identification by LC-MS/MS

LC-MS/MS analyses were carried out using a capillary LC system (Waters, UK) coupled to a Q-TOF mass spectrometer (Micromass, Manchester, UK) equipped with a Z-spray ion-source working in the nanoelectrospray mode. Glu-fibrinopeptide was used to calibrate the instrument in MS/MS mode. Tryptic peptides were concentrated and desalted on a 75 μm ID × 150 mm C18 PepMap column (LC Packings, Amsterdam, The Netherlands). Eluent A and B was 0.1% formic acid in 97% water, 3% acetonitrile and 0.1% formic acid in 97% acetonitrile respectively. Six μl of sample were injected into the nanoLC system, and separation was performed using the following gradient: 0 min 7% eluent B, 35 min 50% B, 45 min 80% B, 49 min 80% B, 50 min 7% B, 60 min 7% B. Database search was performed with ProteinLynx screening SWISS-PROT and NCBI. For proteins that were difficult to find, Mascot search tool available on the Matrix Science site screening NCBInr was used.

### Gel scanning and image analysis

Stained gels were scanned using an ImageScanner II (GE Healthcare, Uppsala, Sweden) and ImageMaster™(GE Healthcare, Uppsala, Sweden) was used for computer analysis.

### Flow cytometric analysis of cell surface calreticulin

HL-60 cells were plated in 24-well plates and incubated for the indicated time. Cells were harvested, washed twice with PBS and incubated for 30 min with primary antibody, diluted in cold blocking buffer (2% FBS in PBS), followed by washing and incubation for 30 min with the FITC-conjugated monoclonal secondary antibody diluted 1:500 in blocking buffer. Each sample was then analyzed by FACScan (Becton Dickinson, USA) to identify cell surface calreticulin. Isotype matched IgG antibodies were used as control, and the fluorescence intensity of stained cells was gated on PI-negative cells.

### Western blot analysis

To obtain a cytosolic-rich fraction, ZEA-treated cells were harvested and washed once in ice cold PBS and incubated at 4°C for 10 min with ice-cold cell lysis buffer (250 mM sucrose, 70 mM KCl, 0.25% Triton X-100, 100 μM PMSF, 1 mM DTT in PBS with complete mini protease inhibitor cocktail). The cell suspension was centrifuged at 20,000 × *g *for 20 min. The supernatant was collected as the cytosolic-rich fraction. Protein concentration of the cytosolic-rich fraction was determined by the Bradford method. Cytosolic proteins (50 μg) were separated by 17% SDS-PAGE and transferred onto nitrocellulose membranes. After treating with 5% non-fat milk in TBS containing 0.2% Tween-20 (blocking buffer), membranes were incubated with mouse monoclonal antibodies to cytochrome c, Bax and Bcl-2 and rabbit polyclonal antibody to Bcl-xL. For detection, appropriate horseradish peroxidase (HRP) conjugated secondary antibodies were used at 1:20,000 dilution. Protein bands were visualized on X-ray film with SuperSignal West Pico Chemiluminecent Substrate.

### FACS analysis for cytosolic and mitochondrial Ca^2+ ^levels

Cytosolic Ca^2+ ^levels were determined using the fluorescence dye 1 μM Fluo3-AM in FITC setting. Mitochondrial Ca^2+ ^levels were determined using the fluorescent dye 250 nM Rhod2-AM in PE setting. After treatment with ZEA for 4 h, cells were incubated with fluorescent dye for 15 min at 37°C, and washed with PBS containing 10 mM glucose and analyzed immediately by flow cytometry. In each analysis, 10,000 events were recorded and analyzed by FACScan (Becton Dickinson, USA).

### RNA extraction and gene expression analysis

Real-time PCR was used to examine expression of endoplasmic reticulum stress genes, viz. calreticulin (CRT), glucose-regulated protein-78 (GRP78) and endoplasmic reticulum protein-29 (ERp29), in the human leukemic cell culture. RNA was isolated from HL-60 cell culture using RNA extraction kit following the manufacturer's protocol. Total RNA (1 μg) was converted to cDNA using RevertAid™ First Strand cDNA Synthesis Kit. For determination of ER stress gene expression, SYBR Green detection was used and the values were normalized using glyceraldehyde-3-phosphate dehydrogenase (GAPDH). Real-time quantitative polymerase chain reaction (PCR) was performed in a DNA Engine (ABi 7500) using SYBR GREENER qPCR UNIVERSAL. Primers sequences are as in Table 1. Relative expression levels for each primer set were normalized to the expression of GAPDH by the 2^-∆CT ^method [27].

**Table 1:** Primer Sequences Used for Real-time Reverse Transcription Polymerase Chain Reaction.

Gene	Sequences (5'-3')	GenBank accession number
GRP78	Forward: GCCTGTATTTCTAGACCTGCC Reverse: TTCATCTTGCCAGCCAGTTG	NM_005347.3

CRT	Forward: AAATGAGAAGAGCCCCGTTCTTCCT Reverse: AAGCCACAGGCCTGAGATTTCATCTG	NM_004343.3

ERp29	Forward: CCTGAAGATCATGGGGAAGA Reverse: TTCTGGAAGGCAGTCAGGAT	NM_001034025.1

GAPDH	Forward: GAAGGTGAAGGTCGGAGTC Reverse: GAAGATGGTGATGGGATTTC	NM_002046.3

### Statistical analysis

Results were expressed as mean *± *SEM (standard error of mean). Statistical difference between control and treated group was determined by the one-way ANOVA (Kruskal Wallis analysis) at limit of p < 0.05 in triplicate of three independent experiments. For comparison between two groups, data were analyzed using Student's *t*-test.

## Results

### Cell cytotoxicity with apoptotic induction

**Figure 2:**
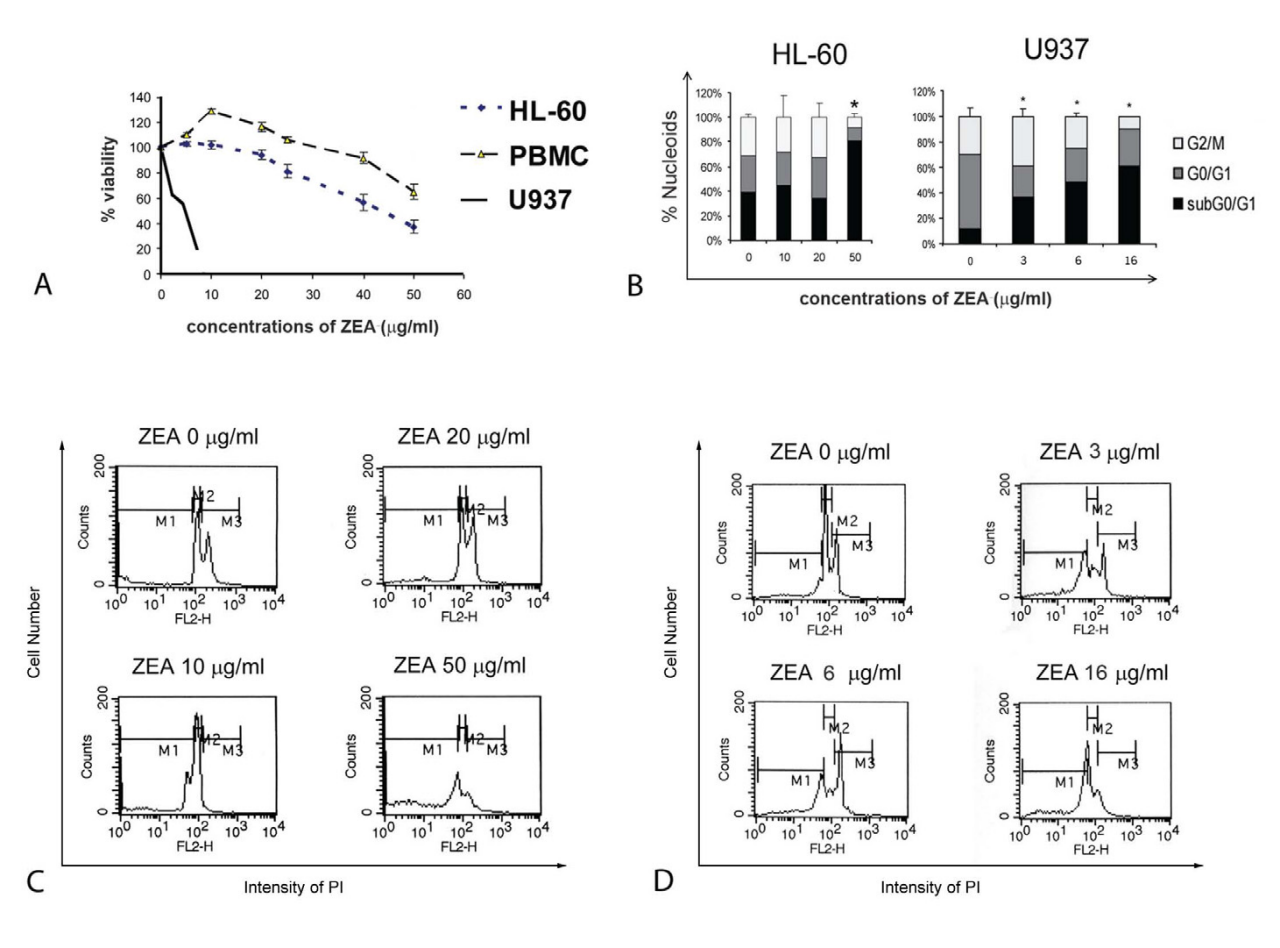
**Effect of ZEA on cell cytotoxicity and apoptotic induction of human leukemic HL-60 and U937 cells**. (A) Cell viability of HL-60, U937 and human peripheral blood mononuclear cells using MTT assay. (B) DNA cell cycle analysis of HL-60 and U937 cells treated with ZEA for 24 h. * p < 0.05, compared with control cells. (C) and (D) Histograms of HL-60 and U937 cells treated with ZEA at indicated concentrations, respectively. Cells were stained with PI and subjected to flow cytometer as described in Materials and methods. M1, subdiploid; M2, G1; M3, G2 M.

Cell viability was evaluated in HL-60, U937 and PBMCs after incubation with ZEA for 24 h using MTT assay. ZEA was toxic to U937 and HL-60 cells with IC_50 _value of 5.1 μg/ml and 44 μg/ml, respectively, but was less toxic to PBMCs, (IC_50 _value > 80 μg/ml) (Figure 2A). However, low concentrations of ZEA (5-20 μg/ml) had a proliferative effect on PBMCs. ZEA induced apoptotic death of HL-60 cells as evidenced by the changes in cell morphology (condensed nuclei and apoptotic bodies) (data not shown) and presence of cells with subdiploid DNA (Figure 2B). There was G1 arrest in HL-60 cells treated with 50 μg/ml ZEA (Figure 2C) and in U937 cells with 16 μg/ml (Figure 2D).

### Mitochondria involvement in ZEA-induced HL-60 and U937 cell apoptosis

**Figure 3:**
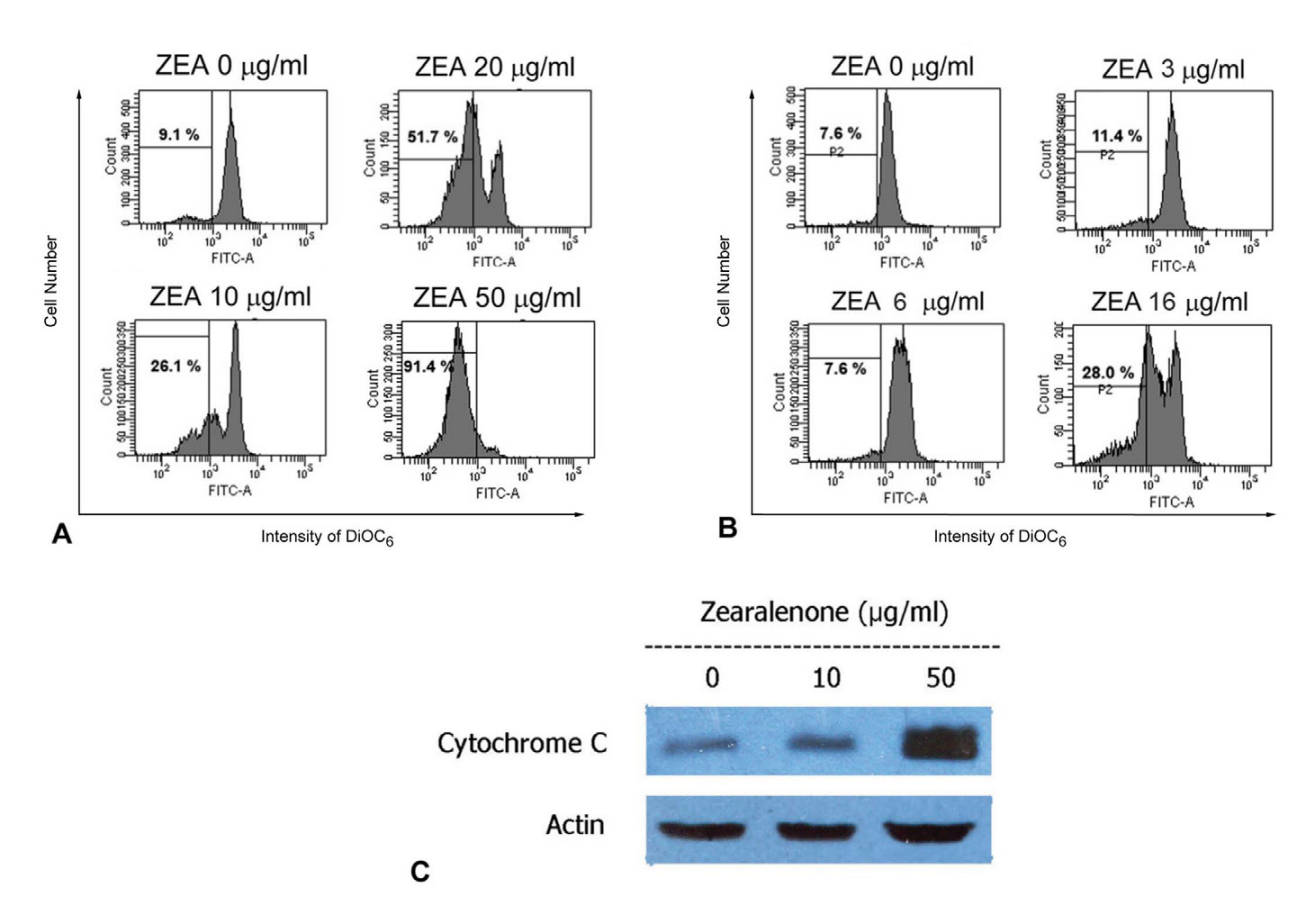
**Mitochondria-mediated human leukemic cell apoptosis**. Reduction of mitochondrial transmembrane potential of HL-60 (A) and U937 (B) cells treated with ZEA. Cells were stained with 40 nM DiOC_6 _for 15 min and then subjected to flow cytometry. Cells with decreased mitochondrial transmembrane potential are less stained with DiOC_6_. (C) Release of cytochrome c from mitochondria. HL-60 cells were treated with ZEA (10, 50 μg/ml) for 4 h and cytosolic cytochrome c was detected by Western blotting. Representative data from three independent experiments are shown.

The reduction of mitochondrial transmembrane potential (MTP) accompanied by release of cytochrome c into cytosol is often associated with apoptosis [28]. Treatment with ZEA resulted in an increase in percent cells with reduced MTP (Figure 3A and 3B) and cytosolic cytochrome c in a dose dependent manner in HL-60 (Figure 3C).

### Expression of Bax, Bcl-2 and Bcl-xL in ZEA-treated HL-60 cells

**Figure 4:**
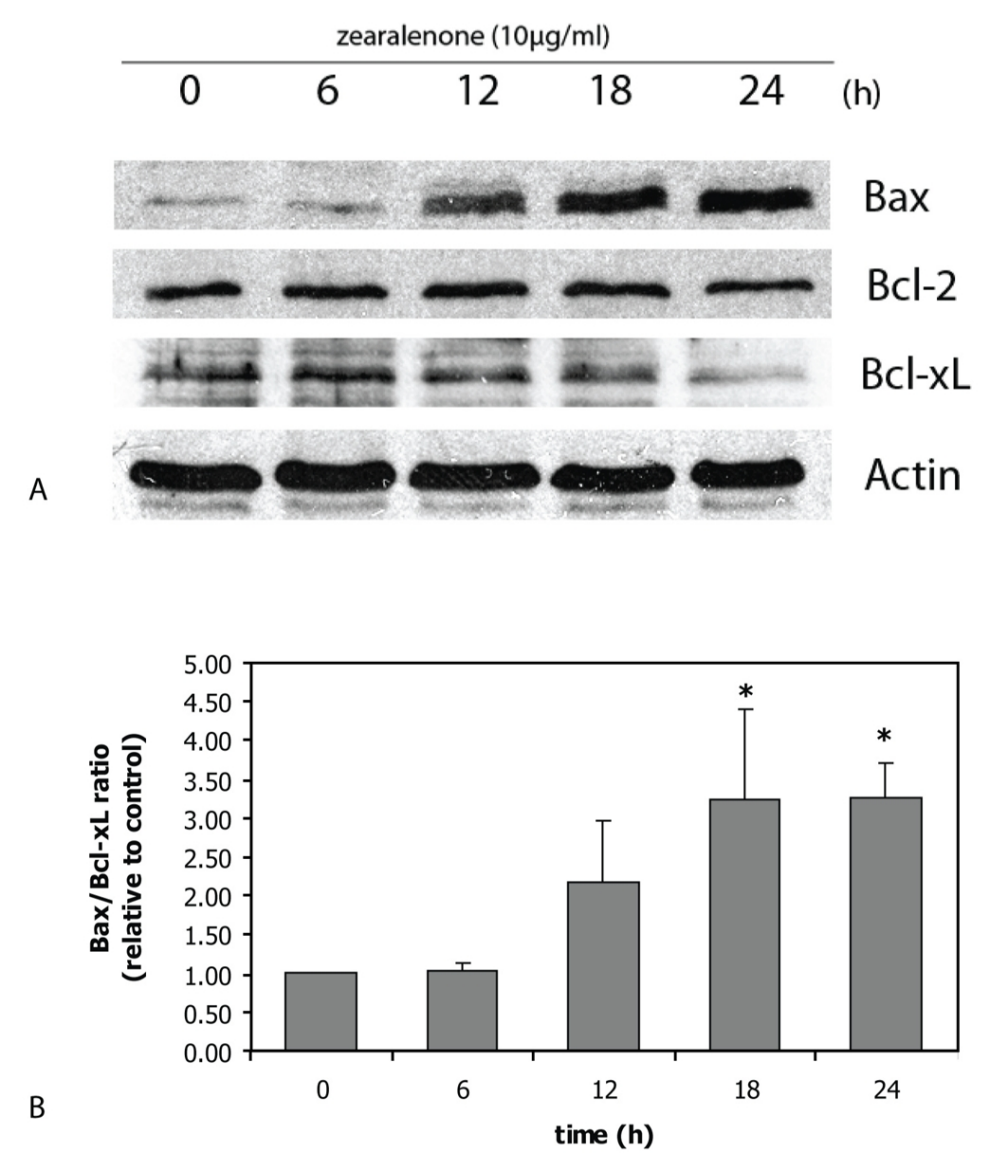
**Expression of Bax, Bcl-2 and Bcl-xL in ZEA-treated HL-60 cells**. Bax, Bcl-2 and Bcl-xL expression (A) and the ratio of Bax/Bcl-xL (B) are from the same sample of cells. Representative data from three independent experiments are shown. The density of bands are plotted as ratio of Bax/Bcl-xL and the results are mean ± S.E.M. from three independent experiments. *, p < 0.05, compared to control.

The mitochondrial apoptotic signaling pathway involves Bax, a proapoptotic Bcl-2 family member, which induces permeabilization of the mitochondrial outer membrane allowing release of cytochrome c [29-31]. Bax expression in HL-60 cells was up regulated in time dependent manner (Figure 4A). Expression of anti-apoptotic Bcl-2 did not change, whereas that of anti-apoptotic Bcl-xL was down regulated time-dependently (Figure 4A and 4B).

### ROS production of ZEA on human leukemic cells

**Figure 5:**
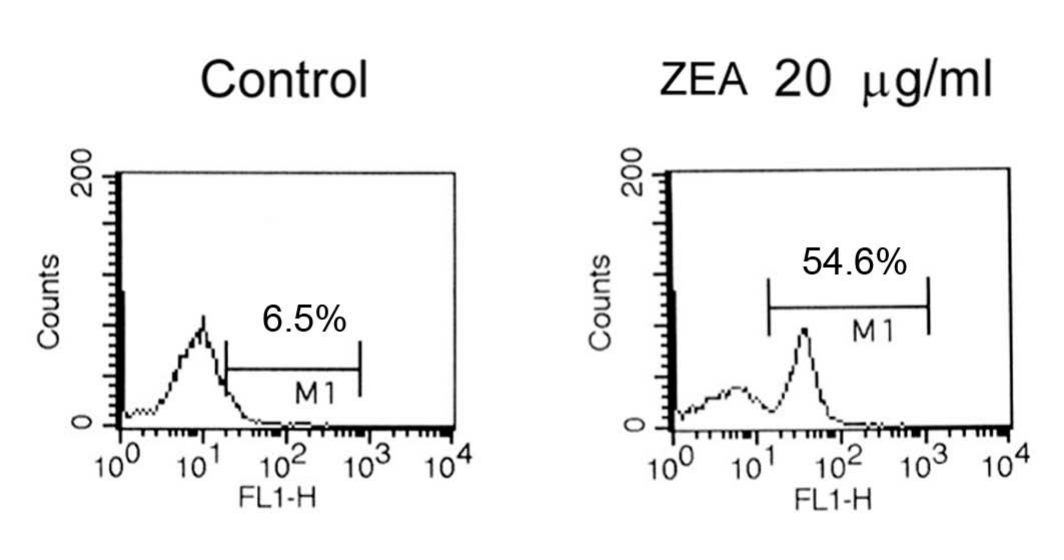
**Generation by ZEA of reactive oxygen species (ROS) in HL-60 cells**. HL-60 cells were treated with 20 μg/ml ZEA for 4 h, incubated with 5 μM DCFH-DA for 15 min and subjected to flow cytometry. Histograms from flow cytometry are shown and cells with increased fluorescence are designated as M1, indicating the presence of ROS.

Changes in MTP are considered to involve ROS production [32]. The ability of ZEA to generate ROS was investigated using a fluorescence sensitive probe (dichlorofluorescein diacetate), which detects peroxide radicals and various other active oxygen radicals [33,34]. ROS was produced in ZEA-treated HL-60 (Figure 5) indicating that the cause of apoptotic cell injury was via oxidative stress.

### Effect of ZEA on activities of caspase-3 and -8 in HL-60 and U937 cells

**Figure 6:**
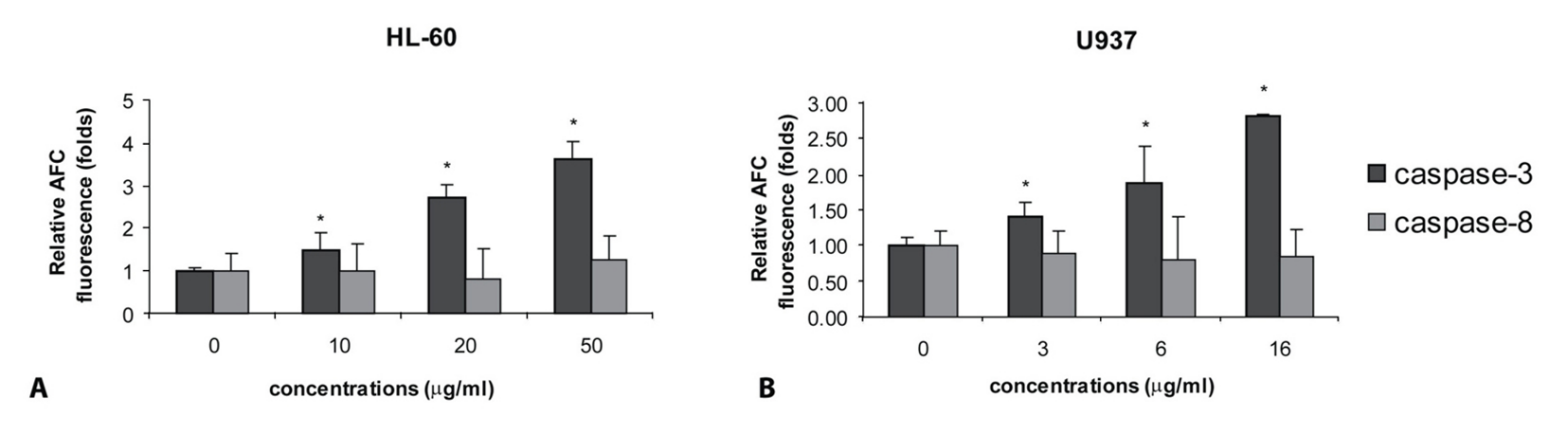
**Effect of zearalenone (ZEA) on activation of caspase-3 and caspase-8**. Activity of caspase-3 and caspase-8 of HL-60 (A) and U937 (B) cells treated for 24 h with various concentrations of ZEA were measured using specific substrate analogs as described in Materials and methods. Data represent mean values ± S.E.M. from three independent experiments. *, p < 0.05, compared to control.

To address the role of activation of caspase activities in ZEA-induced HL-60 and U937 apoptosis, specific caspase substrates were used, namely DEVD-AMC (caspase-3 substrate) and IETD-AMC (caspase-8 substrate). ZEA induced in a dose-dependent manner activation of caspase-3 activity but not that of caspase-8 in HL-60 (Figure 6A) and U937 cells (Figure 6B).

### Protein expression in ZEA-treated U937 and HL-60 cells

**Figure 7:**
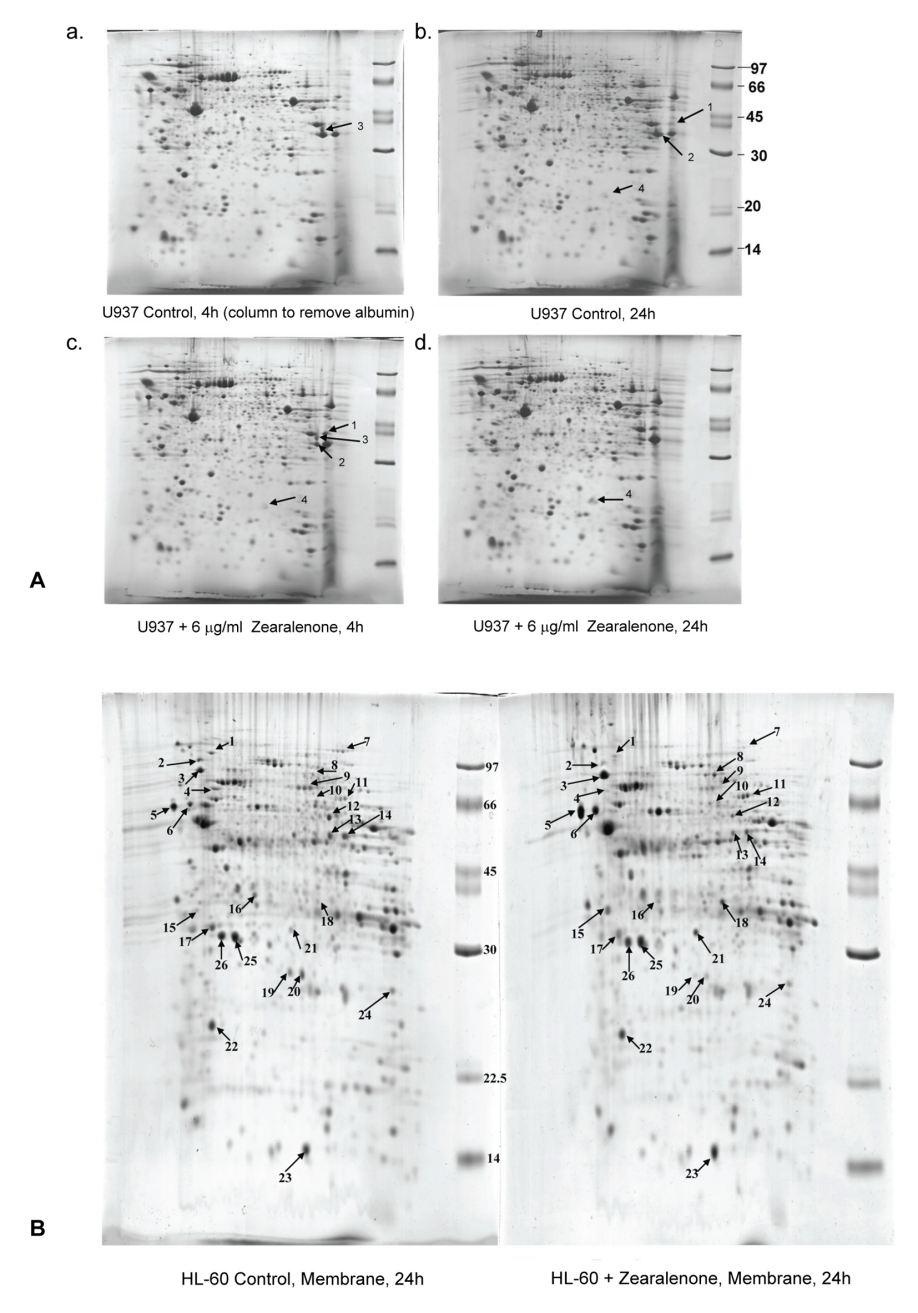
**Two-dimensional polyacrylamide gel-electrophoresis pattern of U937 and HL-60 cells**. (A) U937 cells cultured for 4 and 24 h in the presence or absence of ZEA. (a) control 4 h cells, (b) control 24 h cells, (c) cells treated with 6 μg/ml ZEA for 4 h, (d) cells treated with 6 μg/ml ZEA for 24 h. (Arrow 1) fructose bisphosphate aldolase A, muscle type, lung cancer antigen NY LU1, (arrow 2 and 3) glyceraldehyde 3-phosphate dehydrogenase, (arrow 4) deoxyuridine triphosphate nucleotidohydrolase, mitochondrial precursor. (B) HL-60 cell cultured for 24 h with (right panel) and without ZEA (left panel). There are 22 protein dots of different expression in plasma membrane. The list of proteins identified by LC/MS/MS is shown in Table 1.

The effects of ZEA on protein expression in U937 and HL-60 cells were explored by 2D-PAGE. In U937 cells treated with ZEA for 4 and 24 h, 4 spot differences were detected (Figure 7A), which subsequently were shown by LC-MS/MS to be fructose bisphosphate aldolase A, muscle type, lung cancer antigen NY LU 1 (increased in ZEA-treated cells at 4 and 24 h, arrow 1), glyceraldehyde 3-phosphate dehydrogenase isozymes (GAPDH) (increased in treated cells at 4 and 24 h, arrows 2 and 3) and deoxyuridine triphosphate nucleotidohydrolase mitochondrial precursor dUTP pyrophosphatase (increased in treated cells at 24 h, arrow 4).

2D-PAGE revealed 22 proteins with different expression in the plasma membrane of HL-60 cells treated with ZEA for 24 h compared to control (Figure 7B and Table 2). LC-MS/MS indicated that the up regulated proteins included 78 kDa glucose-regulated protein or GRP78 (Figure 7B and Table 2, dot no. 3; 1.93 folded-increase), calreticulin or CRT (dot no. 5; 2.39 folded-increase), endoplasmic reticulum protein ERp29 (dot no. 21; 2.99 folded-increase), and apoptosis inducing factor (AIF) (dot no. 11; 2.18 folded-increase), whereas expression of heat shock protein 90 (HSP90), which plays a role in ER protein folding [35], was decreased (dot no. 2; 1.55 folded-decrease, Table 2). These results point to the presence of ER stress in ZEA-treated leukemic cells.

**Table 2:** Identified Plasma Membrane Protein Spots in 24 h ZEA-treated HL-60 Cells by LC/MS/MS.

Spot no.	Protein Name(s)	Description	MW/pI	Peptide match	% Coverage	Sequence	*Expression in treated cells (folds)
1	TERA_HUMAN	Transitional endoplasmic reticulum ATPase	89.3/5.18	-	-	-	-1.99

2	GFAP_HUMAN	Glial fibrillary acidic protein	49.8/5.25	1	2.55	(K)LALDIEIATYR(K)	-1.55
			
	K2C8_HUMAN	Keratin, type II cytoskeletal 8	53.7/5.34	1	2.28		
		
	HS90A_HUMAN	Heat shock protein HSP 90-alpha	83.2/4.97	7	10.00	K.IDIIPNPQER.T K.EDQTEYLEER.R K.HFSVEGQLEFR.A R.RAPFDLFENK.K R.GVVDSEDLPLNISR.E K.FYEAFSK.N K.EGLELPEDEEEK.K	

3	GRP78_HUMAN	78 kDa glucose-regulated protein precursor (GRP 78)	72.3/5.10	-	-	-	1.93

4	PLSL_HUMAN	L-plastin, Lymphocyte cytosolic protein 1	70.2/5.02	11	22.01	(K)AACLPLPGYR(V) (K)IGLFADIELSR(N) (R)NEALIALLR(E) (K)LSPEELLLR(W) (K)AYYHLLEQVAPK(G) (R)QFVTATDVVR(G) (K)LNLAFIANLFNR(Y) (R)VNHLYSDLSDALVIFQLYEK(I) (K)FSLVGIGGQDLNEGNR(T) (R)YTLNILEEIGGGQK(V) (K)VNDDIIVNWVNETLR(E)	-3.1

5	CALR_HUMAN	Calreticulin precursor	60.6/4.37	-	-	-	2.39

6	PDIA1_HUMAN	Protein disulfide isomerase precursor	51.1/4.78	-	-	-	2.86

7	EF2_HUMAN	Elongation factor 2	95.1/6.78	-	-	-	-2.87

8	gi|28317	unnamed protein product	59.5/5.17	3	6.00	R.ALEESNYELEGK.I R.QSVEADINGLR.R R.NVQALEIELQSQLALK.Q	2.26

9	DHSA_HUMAN	Succinate dehydrogenase [ubiquinone] flavoprotein subunit, mitochondrial	72.6/7.04	4	8.43	(R)AAFGLSEAGFNTACVTK(L) (R)GVIALCIEDGSIHR(I) (K)NTVVATGGYGR(T) (R)LGANSLLDLVVFGR(A)	-1.29
		
	TCPG_HUMAN	T-complex protein 1 subunit gamma	60.5/6.06	1	2.02	(K)TAVETAVLLLR(I)	

10	SERA_HUMAN	D-3-phosphoglycerate dehydrogenase	56.6/6.28	1	2.44	(K)GTIQVITQGTSLK(N)	-1.34
		
	TCPZ_HUMAN	T-complex protein 1 subunit zeta	58.0/6.22	1	2.26	(K)GIDPFSLDALSK(E)	
		
	gi|4502643	chaperonin containing TCP1, subunit 6A isoform a	58.0/6.23	7	15.00	R.AQAALAVNISAAR.G K.QADLYISEGLHPR.I R.IITEGFEAAK.E K.ALQFLEEVK.V K.SETDTSLIR.G K.GIDPFSLDALSK.E K.VLAQNSGFDLQETLVK.I	
		
	gi|1002923	coronin-like protein	51.0/6.12	7	15.00	R.HVFGQPAK.A R.EPVVTLEGHTK.R R.AVFVSEGK.I K.ILTTGFSR.M R.DAGPLLISLK.D R.AAPEASGTPSSDAVSR.L K.LQATVQELQK.R	

11	119623333	apoptosis inducing factor like isoform CRA d Homo sapiens	63.7/10.23	1	1.21	(R)LLSATSR(T)	2.18
		
	RN112_HUMAN	RING finger protein 112	68.3/8.45	1	1.11	(R)LSGRYPK(V)	
		
	gi|4557014	catalase [Homo sapiens]	59.7/6.90	12	28.00	K.ADVLTTGAGNPVGDK.L K.LNVITVGPR.G K.GAGAFGYFEVTHDITK.Y R.FR.DPILFPSFIHSQK.R STVAGESGSADTVR.D K.NLSVEDAAR.L R.LSQEDPDYGIR.D R.DLFNAIATGK.Y R.LFAYPDTHR.H K.DAQIFIQK.K K.NFTEVHPDYGSHIQALLDK.Y K.NAIHTFVQSGSHLAAR.E	
		
	gi|28317	unnamed protein product	59.5/5.17	7	14.00	R.ALEESNYELEGK.I K.YENEVALR.Q R.QSVEADINGLR.R K.ADLEMQIESLTEELAYLK.K R.NVQALEIELQSQLALK.Q K.QSLEASLAETEGR.Y R.LENEIQTYR.S	

12	SAM50_HUMAN	Sorting and assembly machinery component 50 homolog	51.9/6.46	5	14.50	(K)VNQELAGYTGGDVSFIK(E) (K)EDFELQLNK(Q) (R)THFFLNAGNLCNLNYGEGPK(A) (R)WSYGAGIVLR(L) (R)ICDGVQFGAGIR(F)	-1.98
		
	gi|7022134	unnamed protein product	51.9/6.62	9	20.00	K.DVVVQHVHFDGLGR.T K.VTFQFSYGTK.E R.NFSVNLYK.V K.VTGQFPWSSLR.E K.WEGVWR.E K.VNQELAGYTGGDVSFIK.E K.EDFELQLNK.Q R.FYLGGPTSVR.G R.WSYGAGIVLR.L	
		
	gi|4929571	CGI-51 protein	52.1/6.85	10	26.00	K.DVVVQHVHFDGLGR.T K.VTFQFSYGTK.E R.NFSVNLYK.V K.VTGQFPWSSLR.E K.WEGVWR.E K.VNQELAGYTGGDVSFIK.E K.EDFELQLNK.Q K.QLIFDSVFSASFWGGMLVPIGDKPSSIADRFYLGGPTSIR.G R.FYLGGPTSIR.G R.WSYGAGIVLR.L	
		
	ANX11_HUMAN	Annexin A11	54.3/7.53	5	11.00	R.GTITDAPGFDPLR.D K.TPVLFDIYEIK.E R.LLISLSQGNR.D R.SETDLLDIR.S K.SLYHDISGDTSGDYR.K	

13,14	ENOA_HUMAN	Alpha-enolase	47.0/7.54	-	-	-	-1.57, -1.88

15	119571303	spectrin domain with coiled coils 1 isoform CRA d Homo sapiens	28.9/4.97	1	4.20	(R)LQIVSLASWAR(A)	5.14
		
	ATPG_HUMAN	ATP synthase subunit gamma, mitochondrial	33.0/9.56	1	4.03	(R)IYGLGSLALYEK(A)	
		
	TPM3_HUMAN	Tropomyosin alpha-3 chain	32.8/4.49	1	2.82	(K)HIAEEADR(K)	
		
	ES8L1_HUMAN	Epidermal growth factor receptor kinase substrate 8-like protein 1	80.3/5.66	1	0.69	(K)SGPSR(K)	
		
	gi|16877071	ATP synthase, H+ transporting, mitochondrial F1 complex, gamma polypeptide 1	32.9/9.23	3	11.00	R.IYGLGSLALYEK.A K.HLLIGVSSDR.G K.ELIEIISGAAALD.-	

16	LDHB_HUMAN	L-lactate dehydrogenase B chain	36.6/5.64	2	8.08	(K)SLADELALVDVLEDK(L) (R)VIGSGCNLDSAR(F)	-1.62
		
	AFF4_HUMAN	AF4/FMR2 family member 4	12.7/9.68	1	0.77	(K)NSSSTSKQK(K)	

17	COMT_HUMAN	Catechol O-methyltransferase	30.0/5.12	2	14.02	(K)VTLVVGASQDIIPQLK(K) (K)GTVLLADNVICPGAPDFLAHVR(G)	1.07
		
	PODXL_HUMAN	Podocalyxin like protein 1 precursor	55.6/5.23	1	2.46	(R)LASVPGSQTVVVK(E)	
		
	121944562	immunoglobulin A heavy chain variable region Homo sapiens	11.9/5.64	1	5.50	(K)VDGIEK(Y)	
		
	TRM13_HUMAN	tRNA guanosine-2'-O-methyltransferase TRM13 homolog	54.2/8.01	1	2.49	(R)KTSLETSNSTTK(R)	

18	ANXA1_HUMAN	Annexin A1	38.7/6.63	5	22.00	K.GGPGSAVSPYPTFNPSSDVAALHK.A K.GVDEATIIDILTK.R K.ALTGHLEEVVLALLK.T K.TPAQFDADELR.A K.GTDVNVFNTILTTR.S	3.25
		
	CN102_HUMAN	UPF0614 protein C14orf102	13.2/7.60	1	0.52	(R)LISLAK(C)	

19	SOCS4_HUMAN	Suppressor of cytokine signaling 4	50.6/6.64	1	1.36	(R)SDLAFR(W)	-3.12
		
	K2C1_HUMAN	Keratin, type II cytoskeletal 1(CK-1)	65.8/8.16	4	5.00	R.QFSSR.S K.AEAESLYQSK.Y K.YEELQITAGR.H K.LALDLEIATYR.T	
		
	K2C7_HUMAN	Keratin, type II cytoskeletal 7 (CK-7)	51.2/5.50	1	2.00	K.LALDIEIATYR.K	

20	gi|189054178	unnamed protein product [Homo sapiens]	66.0/7.62	4	6.00	R.SLDLDSIIAEVK.A K.YEELQITAGR.H K.LNDLEDALQQAK.E R.TLLEGEESR.M	-2.84

21	AF047368_1	nebulette Homo sapiens	11.6/7.98	1	0.99	(K)ENQGNISSVK(Y)	2.99
		
	ERp29_HUMAN	Endoplasmic reticulum protein ERp29	29.0/6.77	7	22.00	K.GALPLDTVTFYK.V K.GALPLDTVTFYK.V K.FVLVK.F R.DGDFENPVPYTGAVK.V K.QGQDNLSSVK.E K.WAEQYLK.I K.SLNILTAFQK.K	

22	ATP5H_HUMAN	ATP synthase subunit d, mitochondrial	18.5/5.21	6	40.00	K.TIDWVAFAEIIPQNQK.A K.SWNETLTSR.L R.LAALPENPPAIDWAYYK.A K.AGLVDDFEK.K K.YTAQVDAEEK.E K.YTAQVDAEEKEDVK.S	-1.08
		
	gi|189054178	unnamed protein product	66.0/7.62	3	5.00	K.SLNNQFASFIDK.V R.SLDLDSIIAEVK.A K.LALDLEIATYR.T	

23	B2MG_HUMAN	Beta-2 microglobulin	12.7/5.77	2	18.00	R.VNHVTLSQPK.I K.VEHSDLSFSK.D	1.35

24	NDUBA_HUMAN	NADH dehydrogenase [ubiquinone] 1 beta subcomplex subunit 10	20.8/8.60	3	20.35	(K)AFDLIVDRPVTLVR(E) (K)EVEQFTQVAK(A) (R)YQDLGAYSSAR(K)	-1.05
		
	gi|189054178	unnamed protein product	65.9/7.62	7	12.00	R.TNAENEFVTIK.K R.SLDLDSIIAEVK.A K.YEELQITAGR.H K.LNDLEDALQQAK.E K.LALDLEIATYR.T R.TLLEGEESR.M R.GSGGGSSGGSIGGR.G	

25	ASCC1_HUMAN	Activating signal cointegrator 1 complex subunit 1	45.48/5.22	1	1.75	(R)SFALLPR(L)	1.11
		
	PHB_HUMAN	prohibitin	29.8/5.57	11	52.00	K.FGLALAVAGGVVNSALYNVDAGHR.A K.DLQNVNITLR.I R.FDAGELITQR.E R.AATFGLILDDVSLTHLTFGK.E K.EFTEAVEAK.Q K.QVAQQEAER.A K.AAIISAEGDSK.A K.AAELIANSLATAGDGLIELR.K R.KLEAAEDIAYQLSR.S K.LEAAEDIAYQLSR.S R.NITYLPAGQSVLLQLPQ.-	

26	PHB_HUMAN	Prohibitin	29.8/5.57	13	59.00	K.VFESIGK.F K.DLQNVNITLR.I R.ILFRPVASQLPR.I R.IFTSIGEDYDER.V R.VLPSITTEILK.S R.FDAGELITQR.E R.AATFGLILDDVSLTHLTFGK.E K.EFTEAVEAK.Q K.QVAQQEAER.A K.AAIISAEGDSK.A K.AAELIANSLATAGDGLIELR.K R.KLEAAEDIAYQLSR.S R.NITYLPAGQSVLLQLPQ.-	1.06
		
	NDUS3_HUMAN	NADH dehydrogenase [ubiquinone] iron-sulfur protein 3, mitochondrial	30.2/6.99	2	9.00	K.SLVDLTAVDVPTR.Q K.DFPLSGYVELR.Y	

Note: Spot no. 1, 3, 5, 7, 13 and 14 were matched from our hepatocellular carcinoma cell line database. *The density of spots were calculated as percent volume and shown in this table as folds of increase or decrease (-).

### ER stress gene expression at mRNA levels

**Figure 8:**
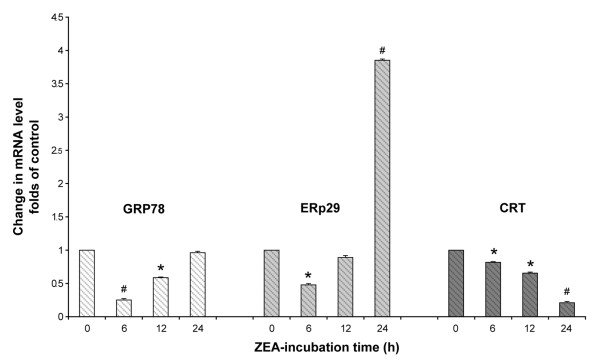
**Real-time reverse transcription polymerase chain reaction of *GRP78*, *CRT *and *ERp29 *genes**. HL-60 cells were treated with 20 μg/ml ZEA for indicated time of incubation. The levels of mRNA were normalized to the level of GAPDH mRNA. After the normalization, the mRNA level was expressed as the fold change compared to that in the basal group untreated with ZEA (at 0 h). Data are the mean ± S.E.M. of three independent experiments. * p < 0.05 compared to control, # p < 0.01 compared to control.

The results of 2-dimensional gel electrophoresis led us to examine the ER stress gene expression at mRNA levels of three genes (*GRP78*, *CRT *and *ERp29*), which were increased in 2-D PAGE (Table 2), employing real-time RT-PCR. GRP78 mRNA had a tendency to be up regulated in a time response manner whereas CRT mRNA was down regulated in a time response pattern as shown in Figure 8. However, ERp29 mRNA expression prominently increased 3.8 folds compared to control (Figure 8), which supported the rising amount of ERp29 protein in 2-D (2.99 folds as in Table 2).

### Cytosolic and mitochondrial Ca^2+^status in ZEA-treated leukemic cells

**Figure 9:**
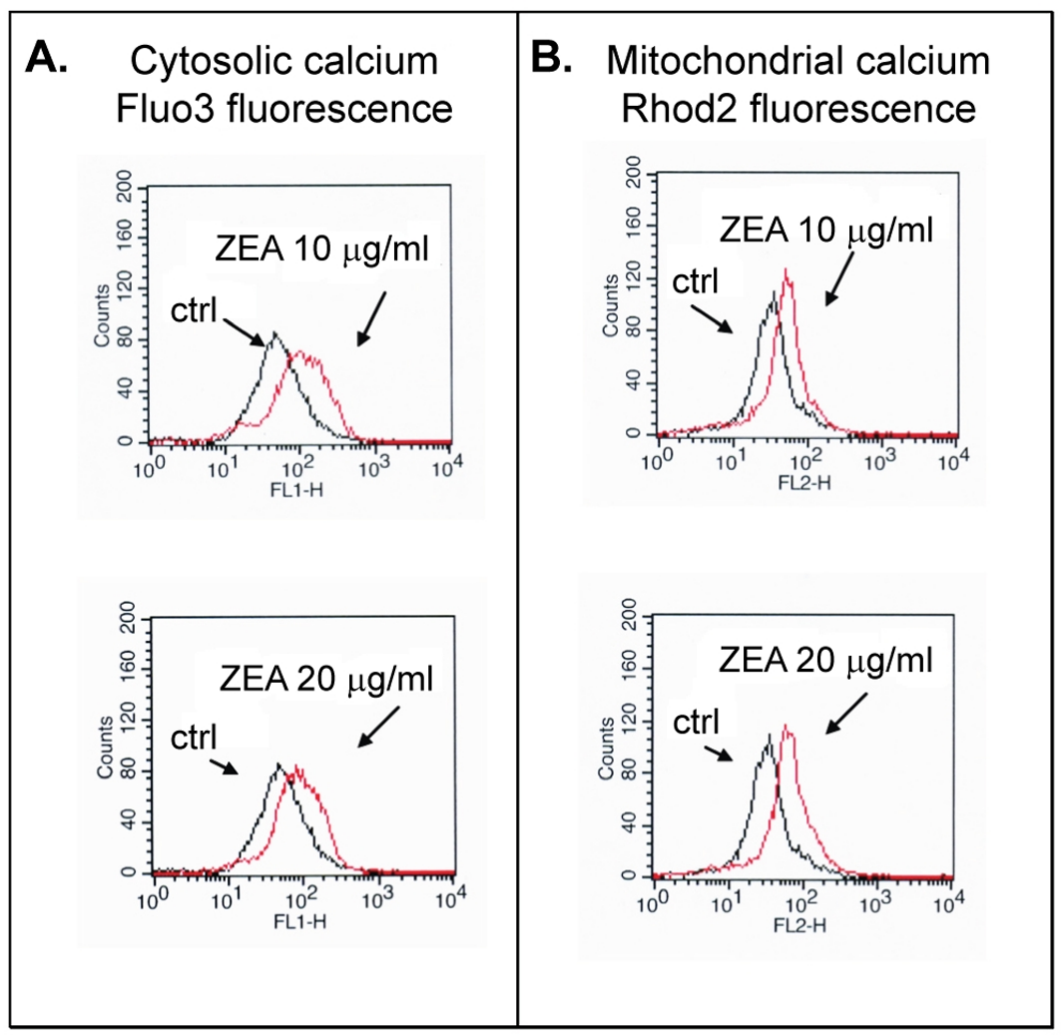
**Effect of ZEA on cytosolic (A) and mitochondrial (B) Ca^2+ ^level in HL-60 cells**. HL-60 cells were incubated with Fluo3 (cytosolic) or Rhod2 (mitochondrial) Ca^2+^- specific dye for 15 min after treatment with and without ZEA for 1 h, then were subjected to flow cytometry as described in Materials and Methods. Black trace, control cells; red trace, ZEA-treated cells. Histogram of FACS analysis represents one of three independent experiments.

Increases in cytosolic and mitochondrial Ca^2+ ^levels have been found in ER stressed cells [36]. As indicated above, apoptosis of leukemic cells induced by ZEA also involved ER stress, Ca^2+ ^levels in both mitochondria and cytosol were measured. FACS analysis histograms of Fluo3-AM-stained (Figure 9A) and Rhod2-stained (Figure 9B) HL-60 cells treated with 10 and 20 μg/ml ZEA revealed increased Ca^2+ ^levels in both cytosolic and mitochondrial compartments.

### Effect of ZEA treatment on calreticulin exposure on cell surface

**Figure 10:**
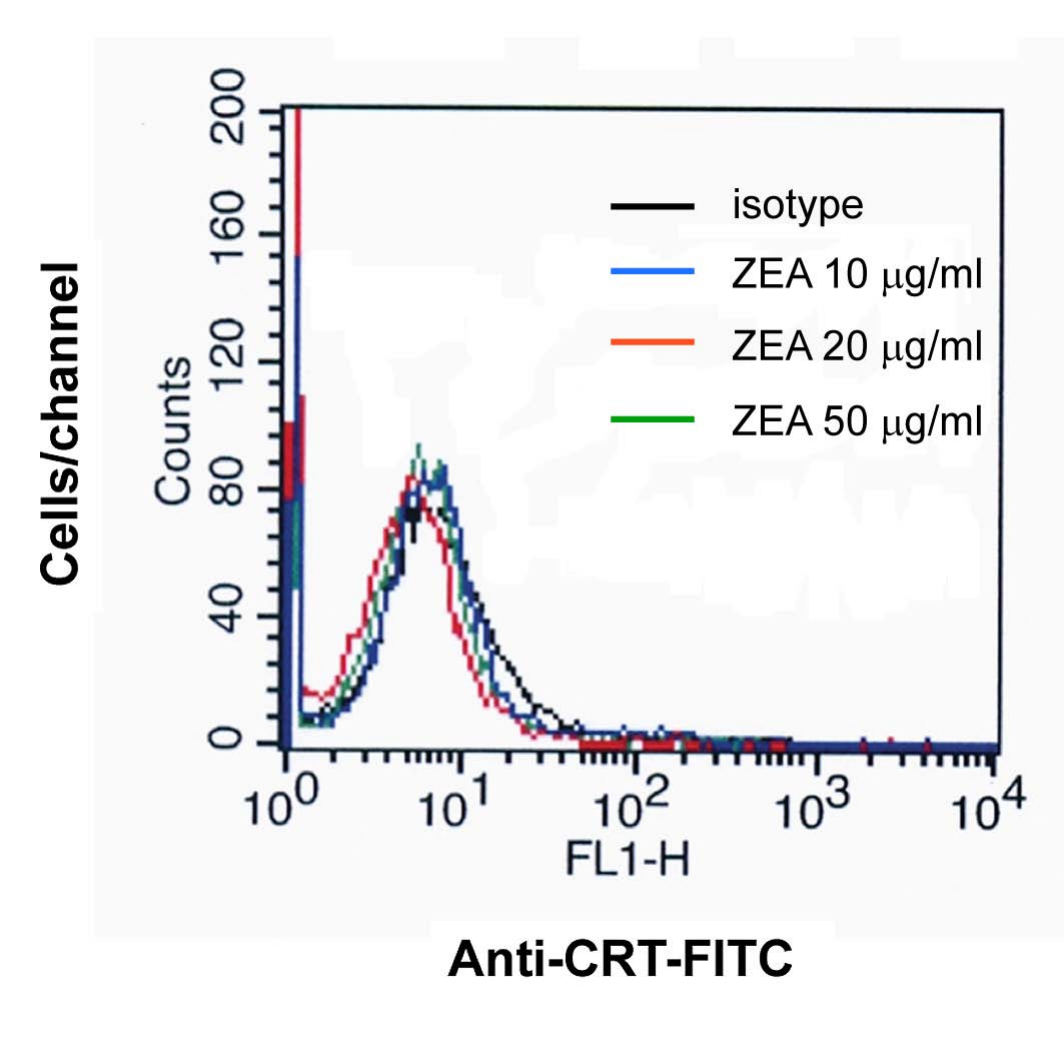
**Cell surface exposure of calreticulin in ZEA-treated HL-60 cells**. Cells were treated for 30 min with ZEA at indicated concentrations and analyzed for the caltreticulin exposure by flow cytometry as described in Materials and Methods. Histogram of FACS analysis represents one of three independent experiments.

Reduction of ER Ca^2+ ^level (ER stress) favors cell surface exposure of calreticulin [37]. Exposure for 30 min of HL-60 cells to ZEA (10, 20 and 50 μg/ml) did not produce an increase in the presence of calreticulin on the cell surface as assessed by FACS (Figure 10).

## Discussion

ZEA is a non-steroidal estrogenic mycotoxin produced as a secondary metabolite by several fungi of the genus *Fusarium *[38,39]. In the present study, ZEA induced apoptosis in human leukemic HL-60 and U937 cell lines, but less in PBMCs, as evidenced by presence of apoptotic bodies and cells with subdiploid peaks (representing DNA fragmentation). ZEA is cytotoxic to bovine lymphocytes [40] and induces human PBMC apoptosis and necrosis depending on the concentrations of ZEA [41].

Two central pathways have been shown to be involved in the process of apoptotic cell death: one is the death receptor pathway with direct involvement of caspase-8 and the other is the mitochondrial pathway in which cytochrome c is released from mitochondria into cytosol. Data presented here suggest that mitochondrial dysfunction is the mechanism involved in ZEA-induced apoptotic death in human leukemic cells. ZEA targets mitochondria and/or lysosomes and induces lipid peroxidation (indicating oxidative stress) and cell death in human colon Caco-2 cell line [42]. The loss of mitochondrial transmembrane potential and the increase of ROS generation were early events caused by ZEA. The following two possibilities are proposed: (i) ZEA increases ROS production which leads to mitochondrial dysfunction; (ii) Mitochondrial dysfunction is induced by ZEA treatment and results in ROS generation.

Bax, a pro-apoptotic protein in Bcl-2 family, was upregulated indicating the involvement of mitochondria, as Bax forms channels at the outer mitochondrial membrane to facilitate the release of cytochrome c [43,44]. Activation of mitochondrial permeability transition is required for the complete release of cytochrome c [45,46]. The increased ratios of Bax/Bcl-2 and Bax/Bcl-xL in ZEA-treated human leukemic cells would facilitate this process. It has been recently reported that ZEA-induced human hepatoma HepG2 cell apoptosis also involves mitochondrial alterations including Bax relocalization into the mitochondrial outer membrane, loss of mitochondrial transmembrane potential, permeability transition pore complex opening, ROS production and cytochrome c release [32].

Proteomic profiling of ZEA-treated and untreated U937 cells revealed a role of enzymes in carbohydrate and nucleotide metabolism in apoptosis. Besides its role in glycolysis, GAPDH initiates a cell death cascade [47]. Diverse apoptotic stimuli activate inducible nitric oxide synthase (iNOS) or neuronal NOS (nNOS), with the NO S-nitrosylating GAPDH, abolishing its catalytic activity and conferring on it the ability to bind to Siah1, an E3-ubiquitin-ligase with a nuclear localizing signal. The GAPDH-Siah1 protein complex, in turn, translocates to the nucleus and mediates cell death.

The involvement of ER stress in ZEA-induced apoptosis shown in this study led to an investigation of CRT, an ER-resident stress-regulated chaperone with C-terminal KDEL signal [48,49]. Under certain circumstances, ER dysfunction leads to an accumulation of unfolded or misfolded proteins in the ER lumen and activates compensatory mechanism, which has been referred to as ER stress response or unfolded protein response [50]. Several ER transmembrane proteins are identified as sensors of ER stress. These include pancreatic ER kinase (PERK), inositol requiring enzyme 1 (IRE1) and activating transcription factor 6 (ATF6). PERK phosphorylates the alpha subunit of eukaryotic initiation factor 2 (eIF2alpha), which attenuates the initiation of translation in response to ER stress. The activation of IRE1 and ATF6 signaling promotes pro-apoptotic transcription factor CHOP and the expression of ER-localized chaperones, such as CRT, GRP78 and GRP94, which facilitate the restoration of proper protein folding within the ER [50]. These protective responses result in an overall decrease in translation, enhanced protein degradation and increased levels of ER chaperones, which consequently increase the protein folding capacity of the ER. However, sustained ER stress ultimately leads to decreased ER chaperone and cell death [50]. CRT was translocated to the cell membrane of human leukemic cells treated with ZEA (Figure 7B). ER also regulates calcium ion homeostasis and Ca^2+ ^levels were increased in cytosol and mitochondria, suggesting the involvement of ER stress in ZEA-treated human leukemic cells. 2D-PAGE of HL-60 treated cells showed increased expression of GRP78, ERp29 and CRT precursor confirming the existence of ER stress. Real-time reverse transcription PCR supported the involvement of ERp29 in the human leukemic HL-60 cell apoptosis. For CRT and GRP78 gene expression, the mRNA might not be stable and was degraded at the measured-time. Nevertheless, ER stress can also activate caspase-9 by releasing cytochrome c from mitochondria to cytosol [24,25].

The accumulation of unfolded proteins in the ER was a marker of cellular stress induced by ZEA. Oxidative stress was also found in ZEA-stimulated human leukemic cell apoptosis (Figure 5). The involvement of ER stress and oxidative stress in ZEA-induced apoptosis of human leukemic cell lines are first described, however, further experiments are required to demonstrate the signaling relationship between the oxidative stress and ER stress.

The contents of ZEA in the daily intake might enhance the apoptotic effect of promyelocytic and monocytic leukemic cell lines in the leukemic patients. ZEA-induced apoptosis and necrosis occur in human PBMCs *in vitro *depending on the concentrations of ZEA [41]. The major metabolites of ZEA in various species are alpha and beta zearalenol. Alpha and beta zearalenol inhibit cell viability and induce oxidative stress and stress protein (HSP70 and HSP27) expression in Vero cells (kidney epithelial cells extracted from African green monkey) [51]. However, more studies should be performed in *in vivo *model before using ZEA as a therapeutic drug.

Taken together, the intrinsic (mitochondrial) and ER stress pathways cooperated in ZEA-induced human leukemic cell apoptosis. An understanding of the mechanism of ZEA-activated leukemic cell death is a basic step in clinical therapeutic approaches.

## Competing interests

The authors declare that they have no competing interests.

## Authors' contributions

RB, OK and PK conceived, designed and implemented the study, and drafted the manuscript. The 2-D PAGE coupled with LC-MS/MS analysis were performed and supervised by DC, PS, CS and JS. All authors read and approved the final draft of the manuscript.
